# Removal of metal ions and humic acids through polyetherimide membrane with grafted bentonite clay

**DOI:** 10.1038/s41598-018-22837-1

**Published:** 2018-03-16

**Authors:** Raghavendra S. Hebbar, Arun M. Isloor, Balakrishna Prabhu, Inamuddin, Abdullah M. Asiri, A. F. Ismail

**Affiliations:** 1 Membrane Technology Laboratory, Department of Chemistry, National Institute of Technology Karnataka, Surathkal, Mangalore, 575 025 India; 2 Department of Chemical Engineering, Manipal Institute of Technology, Manipal University, Manipal, Karnataka 576 104 India; 3 Chemistry Department, Faculty of Science, King Abdulaziz University, Jeddah, 21589 Saudi Arabia; 4 Centre of Excellence for Advanced Materials Research, King Abdulaziz University, Jeddah, 21589 Saudi Arabia; 5 Advanced Membrane Technology Research Center (AMTEC), Universiti Teknologi Malaysia, 81310 Skudai, Johor Bahru Malaysia

## Abstract

Functional surfaces and polymers with branched structures have a major impact on physicochemical properties and performance of membrane materials. With the aim of greener approach for enhancement of permeation, fouling resistance and detrimental heavy metal ion rejection capacity of polyetherimide membrane, novel grafting of poly (4-styrenesulfonate) brushes on low cost, natural bentonite was carried out via distillation-precipitation polymerisation method and employed as a performance modifier. It has been demonstrated that, modified bentonite clay exhibited significant improvement in the hydrophilicity, porosity, and water uptake capacity with 3 wt. % of additive dosage. SEM and AFM analysis showed the increase in macrovoides and surface roughness with increased additive concentration. Moreover, the inclusion of modified bentonite displayed an increase in permeation rate and high anti-irreversible fouling properties with reversible fouling ratio of 75.6%. The humic acid rejection study revealed that, PEM-3 membrane having rejection efficiency up to 87.6% and foulants can be easily removed by simple hydraulic cleaning. Further, nanocomposite membranes can be significantly employed for the removal of hazardous heavy metal ions with a rejection rate of 80% and its tentative mechanism was discussed. Conspicuously, bentonite clay-bearing poly (4-styrenesulfonate) brushes are having a synergistic effect on physicochemical properties of nanocomposite membrane to enhance the performance in real field applications.

## Introduction

Ultrafiltration (UF) has been employed extensively in several membrane filtrations and separation-based technologies, such as the wastewater treatment, chemical and biochemical applications, protein effluent separation, oil-water separation, pollutant rejection etc^1,2^. The morphology (porous structure) and hydrophilicity are the two significant factors to be considered in membrane fabrication processes. A suitable porous membrane material used in the UF process should have excellent mechanical stability, permeability, hydrophilicity and resistance to the feed streams, which play a very vital role in separation process^3^. Polyetherimide (PEI) is a common microfiltration (MF), ultrafiltration (UF), and pervaporation membrane material due to its excellent film-forming capacity, thermal stability, mechanical strength and chemical resistance to wide range of pH^4^. However, pristine PEI membrane hampers their usage to aqua-based media due to its hydrophobic nature. Thus, the modification of polyetherimide is inevitable. The selectivity and performance of polyetherimide membranes can be improvised by employing chemical grafting, surface coating and by blending hydrophilic polymers. Furthermore, apart from the above-mentioned techniques, inorganic materials such as TiO_2_, SiO_2_, Al_2_O_3_, ZnO_2_, Fe_3_O_4_, and LiCl_4_ were also incorporated into the casting solutions for fabricating organic-inorganic hybrid membranes^5–8^. It was found that, by adding certain inorganic additives or fillers will lead to an increased hydrophilicity, pure water flux, and rejection properties. It also has the capacity to improve the mechanical strength, antifouling efficacy, and effective control on physicochemical properties of the membrane surface.

The non-biodegradable nature of heavy metal ions and humic substances in the water causes harm to human health and affect the aquatic ecosystem. Even lower concentrations of these pollutants accumulated in tissues of an organism can cause severe and fatal damage to the health due to their extreme toxicity^9^. For instance, intake of excess copper into the body can lead to severe health problems like convulsions, vomiting, cramps and even death. The accumulation of nickel beyond the permissible limit can cause severe harm to kidneys, lungs, and disorders such as pulmonary fibrosis, gastrointestinal distress, and skin dermatitis. Cadmium has been categorized by the U.S. Environmental Protection Agency as a potential human carcinogen. Prolonged exposure to cadmium causes kidney dysfunction and high levels of exposure can result in death^10^. In another case, the presence of humic substances in water reacts with most commonly used disinfectant and chlorine to form disinfection by-products such as haloacetic acids, trihalomethanes, and other halogenated products. Without an appropriate treatment process, direct exposure to these carcinogenic by-products can lead to cancers, miscarriages and nervous system complications^6^. These examples illustrate that, the presence of heavy metals and humic acid are extremely dangerous, and therefore it is necessary that these substances should be eliminated from water.

Nanocomposite UF membranes could be an attractive alternative in this perspective. The rate of hydraulic permeation and selectivity was enhanced by the inclusion of inorganic additive into base membrane matrix. Predominantly, cost-effectiveness, size and properties of the materials are the key factors to optimize the inorganic additives^9^. Nowadays, naturally available bentonite clays are the new prospective fillers for the polymer composite for water purification applications, which endow with low cost, strong hydrophilicity, net negative charge, facile chemical modification ability and enhances mechanical strength. It is a naturally occurring clay mineral composed of silica tetrahedral sheets and aluminum octahedral sheets. A single unit cell consists of two basic building blocks such as one aluminum octahedral sheet is sandwiched between two tetrahedral silica sheets^10,11^. Panpanit *et al*. investigated the influence of natural bentonite clay in ultrafiltration flux enhancement during the wastewater treatment. This study demonstrated that, the addition of bentonite clay can result in the reduction of total membrane fouling^12^. It has also been confirmed that, bentonite immobilised polymer membrane matrix having the highly porous surface was able to remove hazardous heavy metal ions from the aqueous stream. Also, addition of bentonite has led to an increasing hydrophilicity, porosity, pure water permeation, antifouling resistance capacity, and effective control over the surface properties^13^.

In recent years, polymer brushes grafted on to nanomaterials are viewed as a new type of chemical modification for functionalisation and for improving the membrane physicochemical properties and performances. Polymer brushes are basically assemblies of one end tethered polymer chains at high graft density on a surface of nanomaterials^14,15^. According to Hadjesfandiari *et al*., the properties of polymer brushes are unique and more effective even grafted polymer chains at low graft density^16^. For the grafting of polymer brushes to nanomaterials, distillation-precipitation polymerization is the unique process to prepare surface functionalized nanomaterials (such as sulfonated, carboxylated) without any stabilizing agent or surfactant. Moreover, this method can be adopted easily for scale up due to solvent reflux in the process can impart efficient mixing and oxygen-free environment. In comparison with the typical polymerization methods like group transfer polymerization, radical-polymerization and chain transfer polymerization, distillation-precipitation polymerization offers greater benefits such as, atom economy, lesser reaction time, no metal catalyst, and easy isolation. The mechanism of distillation-precipitation polymerization follows the order of radical initiation of monomer or cross-linker and subsequent chain propagation by chain addition, which increases colloidal stability of modified nanoparticles due to high surface charge^17,18^.

Bai *et al*. described the preparation of negatively charged chitosan membrane by the inclusion of halloysite nanotubes grafted with poly (sodium 4-styrenesulfonate) via distillation-precipitation polymerization method. It has been stated that, degree of grafting was successfully regulated by varying the amount of monomers and the reaction time. The results shown that, hydrophilicity and permeation properties of hybrid membranes were increased considerably^19^. Further, Cai and group members. reported that, Poly (vinylidene fluoride) (PVDF) membranes with covalently immobilized hyper-branched polymers brushes has shown significant improvement in the antifouling and antibacterial properties^20^. The graft copolymers of PVDF was synthesised by using poly[2-(N,N-dimethylamino)ethyl methacrylate] as a side chain, via activators generated by ATRP. Hence, sulfonated polymer brushes grafted over the nanomaterials having the great potential to substantially improve the membrane performance with low fouling propensity.

Based on these observations, poly (sodium 4-styrenesulfonate) was grafted onto the surface of natural bentonite clay via distillation-precipitation polymerisation method, which was employed as negatively charged and hydrophilic additive. Then modified polyetherimide (PEI) membranes were fabricated by phase inversion method by using different amount of the additive dosage with polyvinylpyrrolidone (PVP) as a pore forming agent. The uniform distribution and proper immobilisation of additive in the membrane matrix were confirmed by elemental mapping analysis. The changes in the surface topology and morphological features were observed by SEM and AFM analyses. The resultant membranes were characterised in terms of its water uptake capacity, hydrophilicity, porosity, and permeation ability. The anti-organic fouling properties of the membranes were examined by using BSA protein molecules solution and humic acid rejection capacity was also studied in detail. Additionally, the detrimental heavy metal ion rejection capacity of the hybrid membranes was investigated using 100 ppm of cadmium nitrate and lead nitrate solution.

## Materials and Methods

### Materials

The (Methacryloxy)propyltrimethoxysilan (MPS), styrene (St), Sodium-p- styrene sulfonate (SS) and 2,2′- Azobisisobutyronitrile (AIBN) were obtained from Alfa Aesar. Polyetherimide (PEI) with melt index 9 g/10 min and Bentonite clay were procured from Sigma Aldrich (India) (S-1). The humic acid was procured from Himedia, India. BSA was obtained from Central Drug House (CDH), India. N-methyl pyrrolidone (NMP) was procured from Merck, India. The lead nitrate and cadmium nitrate were purchased from Sigma Aldrich (India). Deionised water was used throughout the experiment.

### Grafting of sodium4-styrenesulfonate brushes onto bentonite

**Figure 1:**
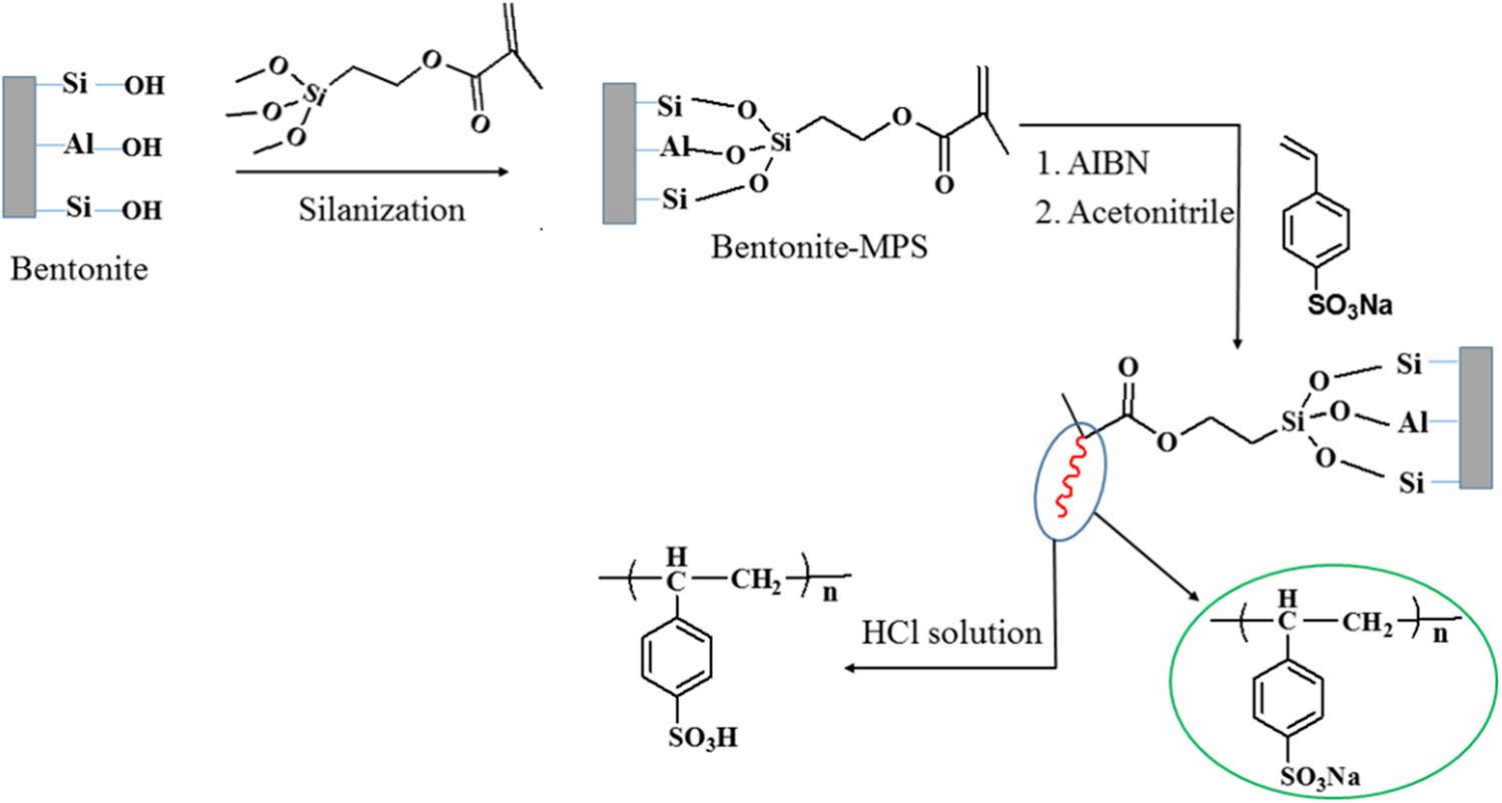
Preparation process of modified bentonite.

The grafting of sodium 4-styrenesulfonate on bentonite was carried out according to distillation precipitation polymerisation method^21^. The schematic representation of the reaction was represented in the Fig. 1. In brief, the bentonite (3 g) was dispersed into the mixture of water (90 ml), ethanol (9 ml) and an aqueous solution of ammonia (7.5 ml). The resultant suspension was magnetically stirred for 24 hrs at room temperature. To this suspension, MPS (0.6 ml) was added dropwise and being stirred for another 24 hrs. Then MPS modified bentonite was collected by centrifugation and dried in an oven at 50 °C. MPS-modified bentonite (0.60 g), St (0.80 ml) SS (0.80 ml), and AIBN (0.032 g) are dispersed into acetonitrile (160 ml) in a dried three-necked round bottom flask. The suspension was heated and kept under boiling condition until half acetonitrile was distilled out. The product obtained was collected by centrifugation and purified using acetonitrile. The resultant hybrid bentonite was then treated with 0.1 M HCl to exchange Na^+^ in –SO_3_Na with H^+^. Then bentonite grafted with poly (4-styrenesulfonate) brushes (modified bentonite) was obtained after being dried in the vacuum oven at 70 °C.

### Membrane preparation

The PEI/ modified bentonite nanocomposite membranes with different amount of additive concentration were prepared by immersion precipitation method^22^. Firstly, modified bentonite was dispersed in a calculated amount of NMP and it was ultra-sonicated for 20 min to confirm uniform dispersion. To the dispersed solution, the desired amount of PEI and PVP (invariable pore former) were added and gently stirred for 18 h to get homogeneous polymer solution. Thus obtained solution was degasified by ultra-sonication to eradicate trapped air bubbles. The homogeneous casting solution was cast over a glass plate and immersed in the coagulation bath containing water as non-solvent. The resulting membrane was kept in coagulation bath for 24 h to ensure the complete phase inversion. The overview of the composition of casting solution was presented in Table 1.

**Table 1:** Composition of casting solutions.

Membrane	PEI (g)	NMP (g)	Ben-(SPB) (g)	PVP (g)	W* (wt%)
PEM-0	16.5	78.5	0	4	0
PEM-1	16.5	78.3	0.16	4	1
PEM-2	16.5	78.1	0.33	4	2
PEM-3	16.5	78.0	0.49	4	3

*W means mass ratio of modified bentonite to PEI.

### Characterization of modified bentonite

The changes in the functional groups after the modification was confirmed by Fourier transform infrared (FTIR) spectroscopy within range 4000–400 cm^−1^ (Bruker FTIR instrument). In order to investigate the elemental composition of nanoparticles before and after modification, TEM (JEM 1230 Electron Microscope), SEM, and EDX (Joel JSM-6380LA scanning electron microscope instrument) analysis were performed. The surface modification of bentonite was characterized by X-ray diffraction (XRD) using Cu-Kα radiation over the 2θ range of 20−60° at a scan rate of 2 deg min^−1^ (Bruker AXS Diffractometer D8 powder XRD). The percentage weight loss before and after the modification was carried out by TGA measurements, using Perkin Elmer (TGA4000/pyris6) instrument in the temperature range of 35 °C −800 °C.

## Membrane Characterization

### Water uptake study

The wettability or water adsorption capacity of prepared nanocomposite membrane was determined as per literature^23^. In brief, immersing the membranes (2 cm^2^) in demineralized water for about 24 h. The weight of the wet membrane was noted after wiping the surface water. The obtained membrane was dried in hot vacuum oven at 75 °C for a 48 h and dry weight was noted. Further water content of the resultant membrane was calculated according to the equitation

1 $$ \% \,uptake=(\frac{{W}_{w}-{W}_{d}}{{W}_{w}})\times 100$$

where *W*_*W*_ and *W*_*d*_ are the weights of the wet membrane and dry membrane respectively.

### Morphological study of the membrane

The variation in cross sectional morphology of PEI membrane after the addition of modified bentonite additive into membrane matrix was analysed by SEM (Joel JSM-6380LA scanning electron microscope) analysis. Prior to SEM analysis, the dry membrane was cryogenically fractured in liquid nitrogen environment and a thin layer of gold was coated using a sputtering apparatus.

### Contact angle measurement and porosity analysis

The contact angle measurement indicates the influence on membrane hydrophilicity. For this purpose, FTA200 Dynamic Contact Angle Analyzer was used and sessile droplet method was followed. From these values, the nature of adhesion or surface energy (ωA) of the membranes could be determined as

2 $${\omega }_{A}={\gamma }_{w}\,(1+cos\theta )$$

where ‘*ω*_*A*_’ is the surface energy (N/m), ‘*γ*_*w*_’ is the surface tension of water (7.2 × 10^−2^ N/m) and ‘*θ*’ is the contact angle.

The presence of polar functional groups such as –OH, –SO_3_H and –C=O in the additive offers considerable changes in the porosity of the resultant membrane. It was determined according to dry-wet weight method^24^. The porosity of membrane was calculated using equation (3)

3 $$P( \% )=\frac{{W}_{w}-{W}_{d}}{{\rho }_{w}\times A\times \delta }\,\times 100\,$$

where ‘*P*’ is the porosity of membrane, ‘*P*_*W*_’ is the density of water (0.998 g/cm^3^), ‘*A*’ is the area of membrane (cm^2^) ‘${W}_{w}$’ is the weight of wet membrane (g), ‘${W}_{d}$’ is the weight of dry membrane (g), and ‘*δ*’ is the thickness of membrane (cm).

### Permeation properties

The pure water permeation rate of the nanocomposite membranes was examined by the lab-fabricated dead end UF kit, having a membrane holder of the effective diameter of 5 cm. The prepared nanocomposite membranes were subjected to compaction at 0.45 MPa transmembrane pressure (TMP) for 1 h. The filtration experiments were conducted at room temperature and at 0.4 MPa TMP. The pure water flux was calculated as

4 $${J}_{w}=\frac{Q}{A\times {\rm{\Delta }}t}$$

where ‘*Q*’ is the volume of water (L) permeated through the membrane, *‘J*_*w*_’ (L/m^2^h) is the pure water flux (PWF), ‘*A’* (m^2^) is area of the membrane surface, ‘Δ*t’* (h) is time.

### Antifouling properties

The fouling resistance nature offered by the membranes was determined according to our previously reported work^24^. Before commencing the experiment, each membrane was subjected to compaction for an initial 30 min at 0.45 MPa. Then applied pressure was brought bring down to 0.4 MPa and pure water permeation rate was measured as ‘*J*_*W*1_’ (L/m^2^h). The BSA protein solution with concentration of 0.8 g/L was prepared and filtration experiment was carried out for about 80 minutes. After BSA filtration, again water permeation rate, ‘*J*_*W*2_’ (L/m^2^h) was measured. The antifouling behaviour of the membrane was calculated in terms of flux recovery ratio (FRR) using the equation (5).

5 $$FRR( \% )=(\frac{{J}_{w2}}{{J}_{w1}})\times 100$$

Generally, higher FRR indicates an improved fouling resistance of the membranes. Further to explore the fouling phenomenon following analyses were conducted. To determine the total protein fouling (‘*R*_*t*_’) produced by the membrane after BSA filtration was calculated by equation (6)

6 $${R}_{t}( \% )=(\,\frac{{J}_{w1}-{J}_{p}}{{J}_{w1}})\,\times 100$$

The flux loss caused by both reversible and irreversible protein fouling (‘*R*_*r*_*R*_*r*_’ and ‘*R*_*ir*_’), which were calculated using equation (7) and (8)

7 $${R}_{r}( \% )=(\,\frac{{J}_{w2}-{J}_{p}}{{J}_{w1}})\times 100$$

8 $${R}_{ir}( \% )=(\,\frac{{J}_{w1}-{J}_{w2}}{{J}_{w1}})\times 100$$

where, ‘*J*_*p*_’ is flux during the protein solution filtration,

### Humic acid (HA) rejection study

The 200 ppm of HA solution was employed as feed to investigate the rejection performance of the nanocomposite membranes. The concentration of HA in the feed solution and permeate was measured by a UV-Visible spectrometer (Analytikjena Specord S600) at a wavelength of 254 nm. The percentage of rejection was calculated by using the equitation (9).

9 $$ \% R=(1-\frac{{C}_{p}}{{C}_{f}})\times 100$$

where ‘*C*_*f*_’ and ‘*C*_*p*_’ (mg mL^−1^) are the concentration of the HA in the feed and permeate respectively.

In order to further evaluate the relative permeation rate of nanocomposite membrane, the PWF of the membrane was estimated before studying the fouling resistance offered by HA in contact with the membrane surface. The flux decline was explored in terms of relative fluxes.

### Heavy metal ion rejection study

The heavy metal ion rejection efficacy of nanocomposite membranes was investigated by both ultrafiltration (UF) and polymer enhanced ultrafiltration (PEUF). For PEUF, 100 ppm concentration of lead nitrate and cadmium nitrate solutions were complexed with 1% polyethyleneimine aqueous solution. The prepared solution was filled into a feed tank and pressurized as required using a nitrogen cylinder and permeate was collected for a specific interval of time. The concentration of heavy metal ions in the feed and permeate was determined using AAS instrument. The percentage of rejection was calculated by using the equation (10)

10 $$ \% R=(1-\frac{{C}_{p}}{{C}_{f}})\times 100$$

where *C*_*f*_ and *C*_*p*_ concentration feed of and permeate solution respectively.

## Results and Discussion

### Characterisation of modified bentonite and membrane

**Figure 2:**
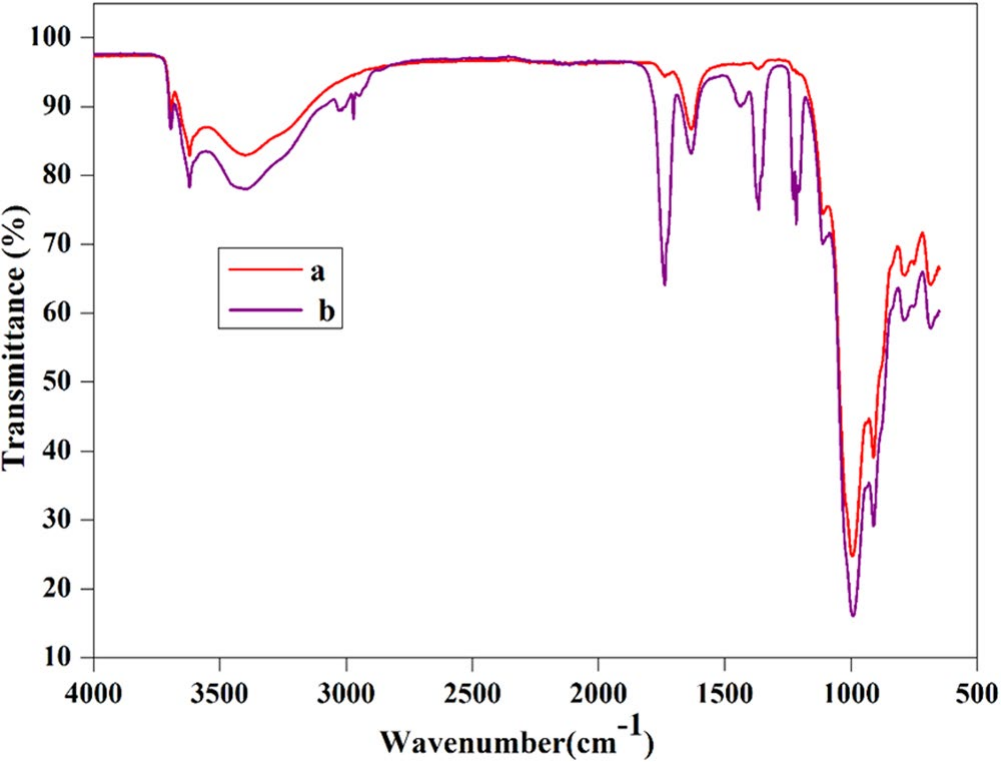
The FT-IR spectrum of (a) bentonite (b) modified bentonite.

**Figure 3:**
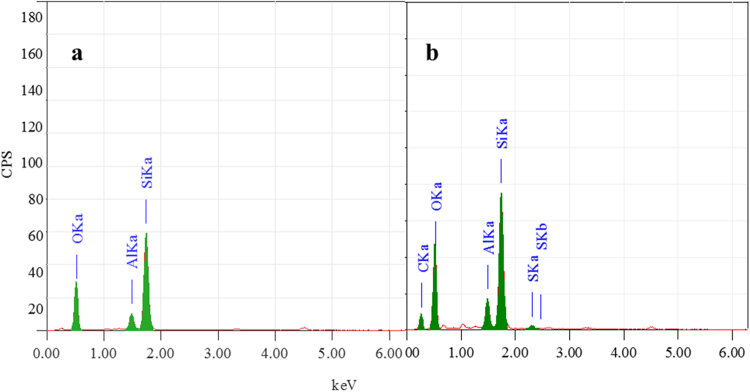
The EDX analysis of (**a**) bentonite and (**b**) modified bentonite.

**Figure 4:**
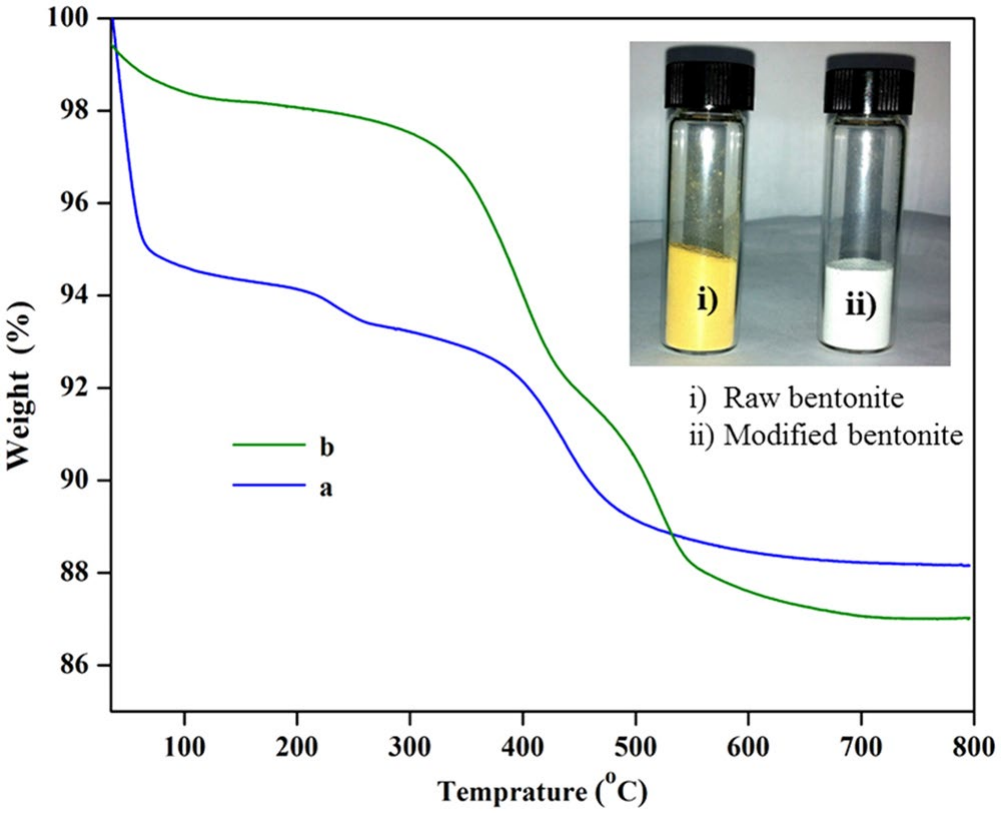
The TGA analysis of (**a**) bentonite and (**b**) modified bentonite.

**Figure 5:**
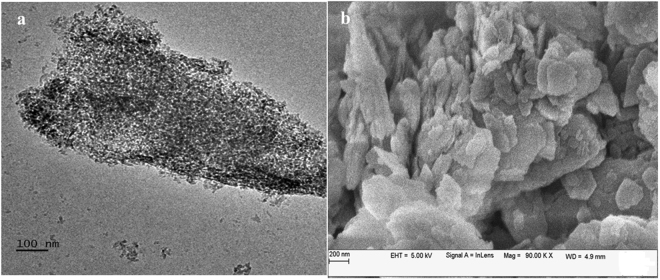
The (**a**) TEM and (**b**) FESEM images of modified bentonite.

**Figure 6:**
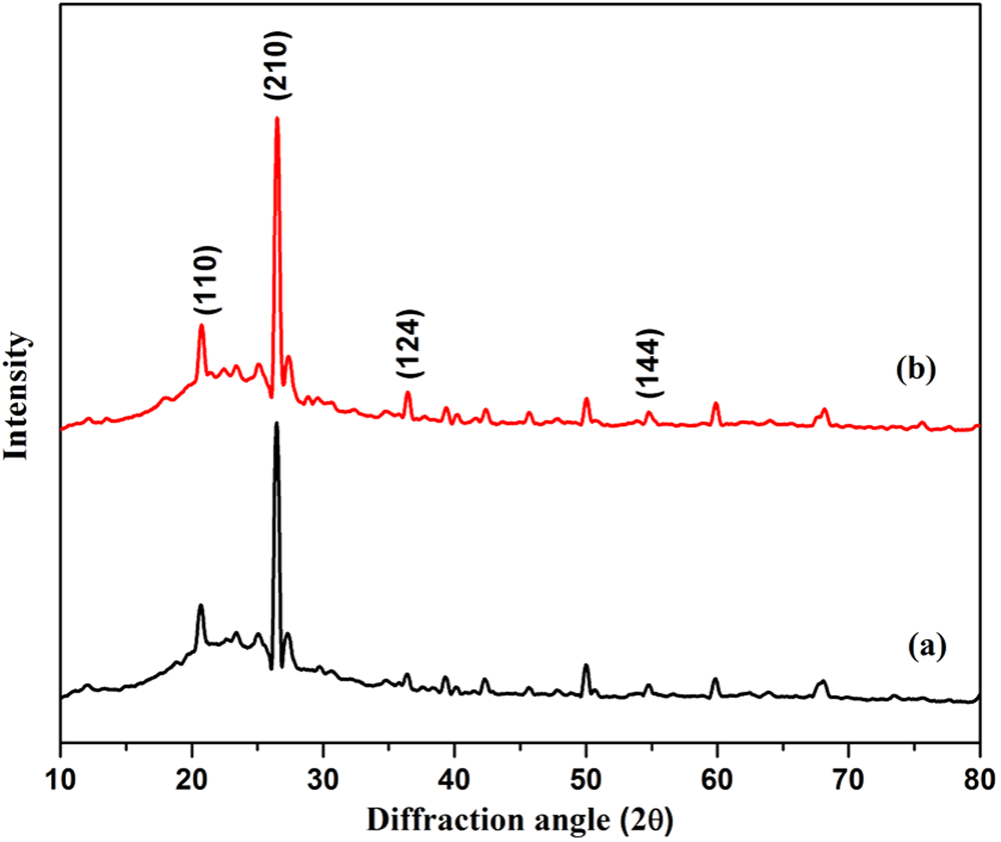
The XRD analysis of (a) bentonite and modified bentonite.

The chemical modification of the bentonite clay was confirmed by taking FTIR spectra of both raw bentonite and modified bentonite (Fig. 2). The spectrum of raw bentonite showed an absorption peak at 3694 cm^−1^ and 3619 cm^−1^ corresponding to the stretching vibrations of hydroxyl groups coordinated to the octahedral cations. The absorption band at 3407 cm^−1^ and 1635 cm^−1^ resultant to the stretching and bending vibrations of –OH functionality of absorbed water molecules on the clay surfaces. The most intensive absorption peak at 998 cm^−1^ attributed to the Si–O stretching vibrations. Compared to raw bentonite, the modified bentonite exhibited new absorption peaks in the spectrum. The characteristic absorption band associated with –CH_2_ stretching vibration was observed at 3010 cm^−1^, peak intensity is low due to low molar concentration. The absorption peak corresponding to the carbonyl (–C=O) group was observed at 1736 cm^−1^. Two new peaks appeared at 1347 cm^−1^ and 1211 cm^−1^ and are ascribed to the stretching vibrations of the sulfonate groups. Furthermore, the intense broad peak for adsorbed water molecules on the bentonite was observed around 3422 cm^−1^. This substantiates the presence of hydrophilic –SO_3_H groups, which enhance the water retention of bentonite^19,21^. In order to further confirm the chemical modification, bentonite and modified bentonite were subjected for energy dispersed x-ray (EDX) analysis. From the Fig. 3, it was observed that, bentonite consists of silicon (Si), aluminium (Al) and oxygen (O) as the main elements. After chemical modification, the peaks corresponding to the carbon (C) with 26.17% and sulphur (S) with 0.27% were appeared along with other peaks. This endorses the chemical modification of the bentonite. The elemental percentage composition of both bentonite and modified bentonite were tabulated in Table 2. In addition to this, TGA analysis was carried out to confirm the chemical modification and resultant output has been presented in Fig. 4. The result revealed that, two-stage weight loss for both bentonite and modified bentonite material. The first-stage of weight loss around 30–200 °C is mainly due to adhered water molecules in the surface and intercalations bentonite structure. The second-stage of weight loss around 350–800 °C is corresponds to the decomposition of organic counterparts present in the material. However, compared to the percentage of weight loss of both the material, modified bentonite exhibited higher weight loss. This is attributed to the fact that, modified bentonite is comprising of attached polyelectrolyte with –SO_3_H functionality and undergo decomposition at a higher temperature^19,21^. Further, the Fig. 5 represents the FESEM and TEM images of the modified bentonite clay (TEM and FESEM images of bentonite ware given in S-2). From the images, it was observed that, bentonite is having a typical layered structure comprising of aluminium and silicon as basic building unit. However, there is no significant structural changes in the bentonite was observed after chemical modification of bentonite (S-1). This was further substantiated by investigating the XRD analysis. The XRD patterns for pure bentonite and modified bentonite nanoparticles are given in Fig. 6. The characteristic peaks at diffraction angle, 2θ = 20.7°, 26.5°, 36.3°, 54.7° corresponds to the planes (110), (210), (124) and (144) of the bentonite material. These XRD patterns are in good agreement with the standard JCPDS file (card no.01-088-0891). From the graph, it can be observed that there is neither significant change in intensity of the patterns, nor there is a shift in the peaks. This behaviour is due to no significant change in the phase structure of material after the modification.

**Table 2:** The elemental composition of the bentonite and modified bentonite.

Samples	Content of element (%)				
	Si	Al	O	S	C
Bentonite	27.63	4.53	67.83	—	—
Modified bentonite	18.80	3.43	51.34	0.27	26.17

### Membrane morphology

**Figure 7:**
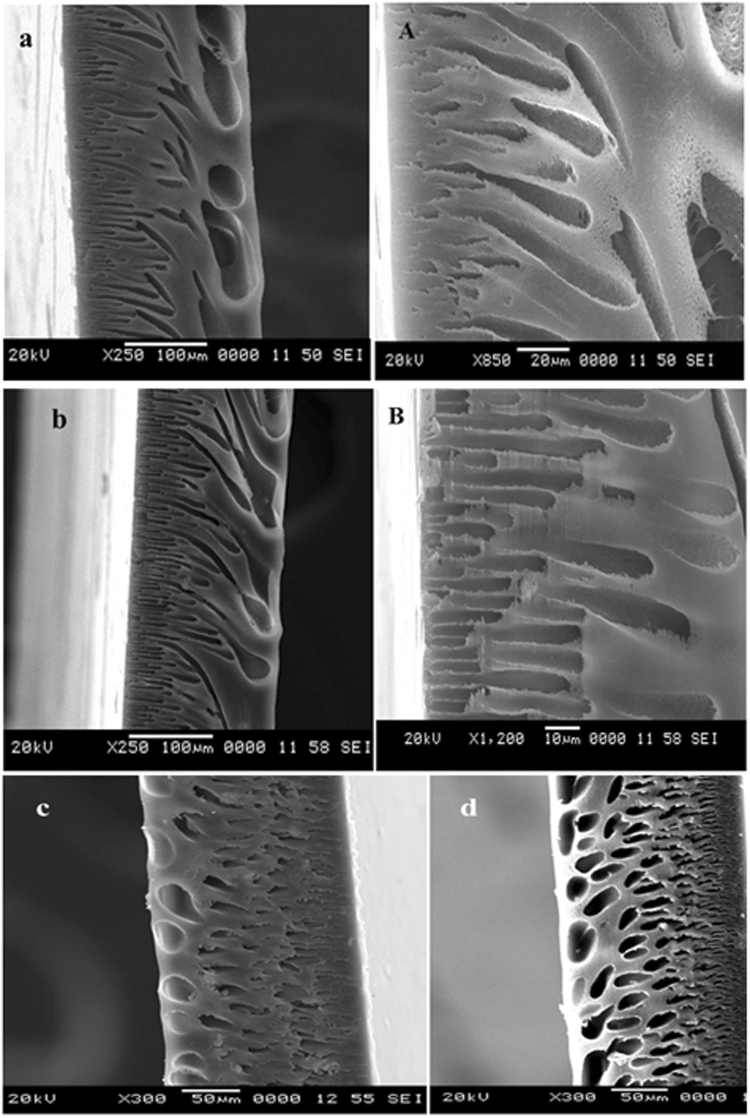
The cross sectional SEM images of the (**a**) PEM-0, (**b**) PEM-1, (**c**) PEM-2 and (**d**) PEM-3 membranes.

**Figure 8:**
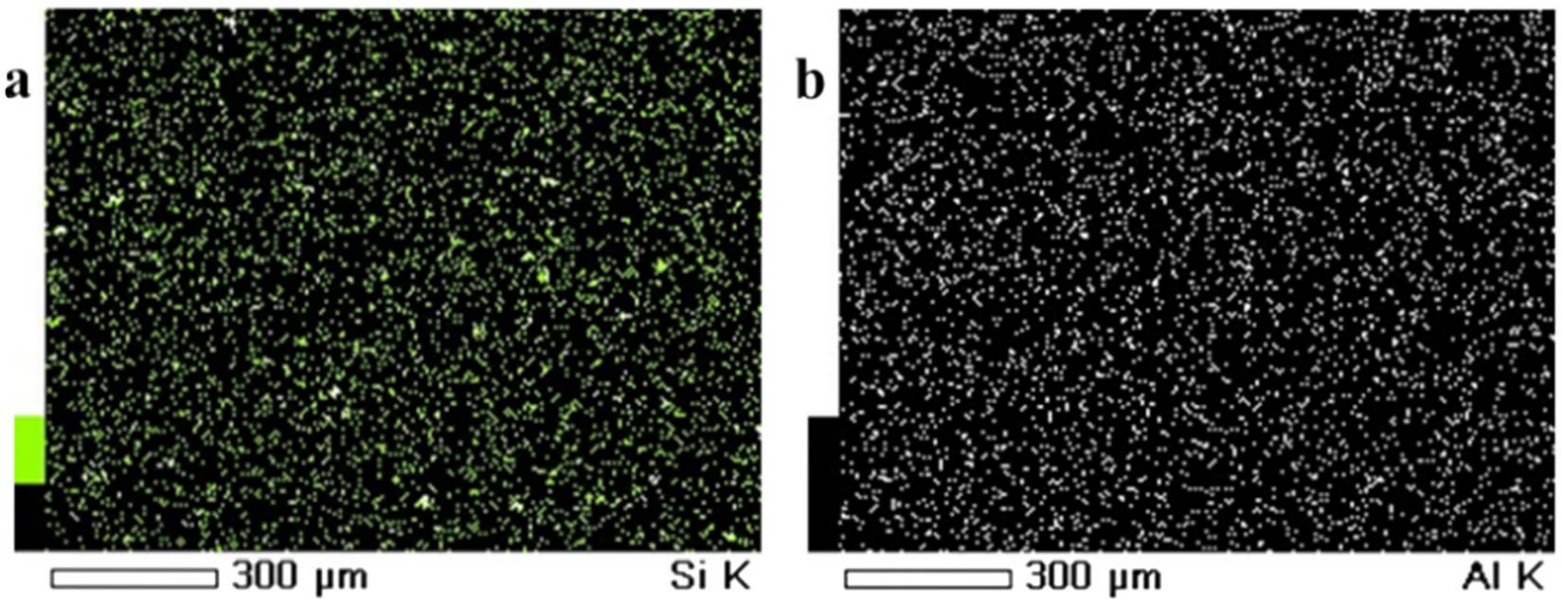
The elemental mapping of (**a**) silicon and (**b**) aluminium of PEM-3 membrane.

In order to examine the influence of modified bentonite content on the internal structure of the membranes, SEM analysis of PEI membranes with different composition have been obtained. The Fig. 7 represents the cross sectional SEM images of nanocomposite membrane matrix with a different amount of additive dosage. All the membranes displayed asymmetric structure with dense top layer followed by porous sublayer with fully developed macro-pores, which is the typical morphology of UF membrane fabricated via the phase inversion process. The proper immobilisation and uniform distribution of the modified bentonite into resultant nanocomposite membrane was confirmed by elemental mapping analysis (Fig. 8). With increasing in the modified bentonite content augmented the number of macrovoid in the sublayer and the pore walls among macro-voids become looser with some channel-like pores. According to the literature, the inclusion of hydrophilic inorganic additives and PVP into the homogeneous casting solution significantly modifies the morphology of resultant membranes^25^. The presence of PVP could expedite the phase inversion process and thus expand the macrovoids. On the other hand, addition of modified bentonite could increase pore interconnection in the membrane matrix because of its migration behaviour. In the current work, changes in morphological features are due to combined effects of both modified bentonite and PVP additives. From the Fig. 5 it was observed that, bentonite is having typical layered structure. According to Ghaemi *et al*., the layered silicate structure of natural bentonite clay and polymer are prone to form intercalated and exfoliated structures. This results in enhancement of the nano-scale interaction between the polymer chains and the modified bentonite additive and at the same time reduced interaction among polymer chains. Moreover, with increasing additive dosage, the increased viscosity of the casting solution hinders the phase inversion phenomenon by delaying solvent and non-solvent exchange rate. Since the growth of the skin layer was reduced, and the formation of macrovoids in the support was improved^26,27^.

**Figure 9:**
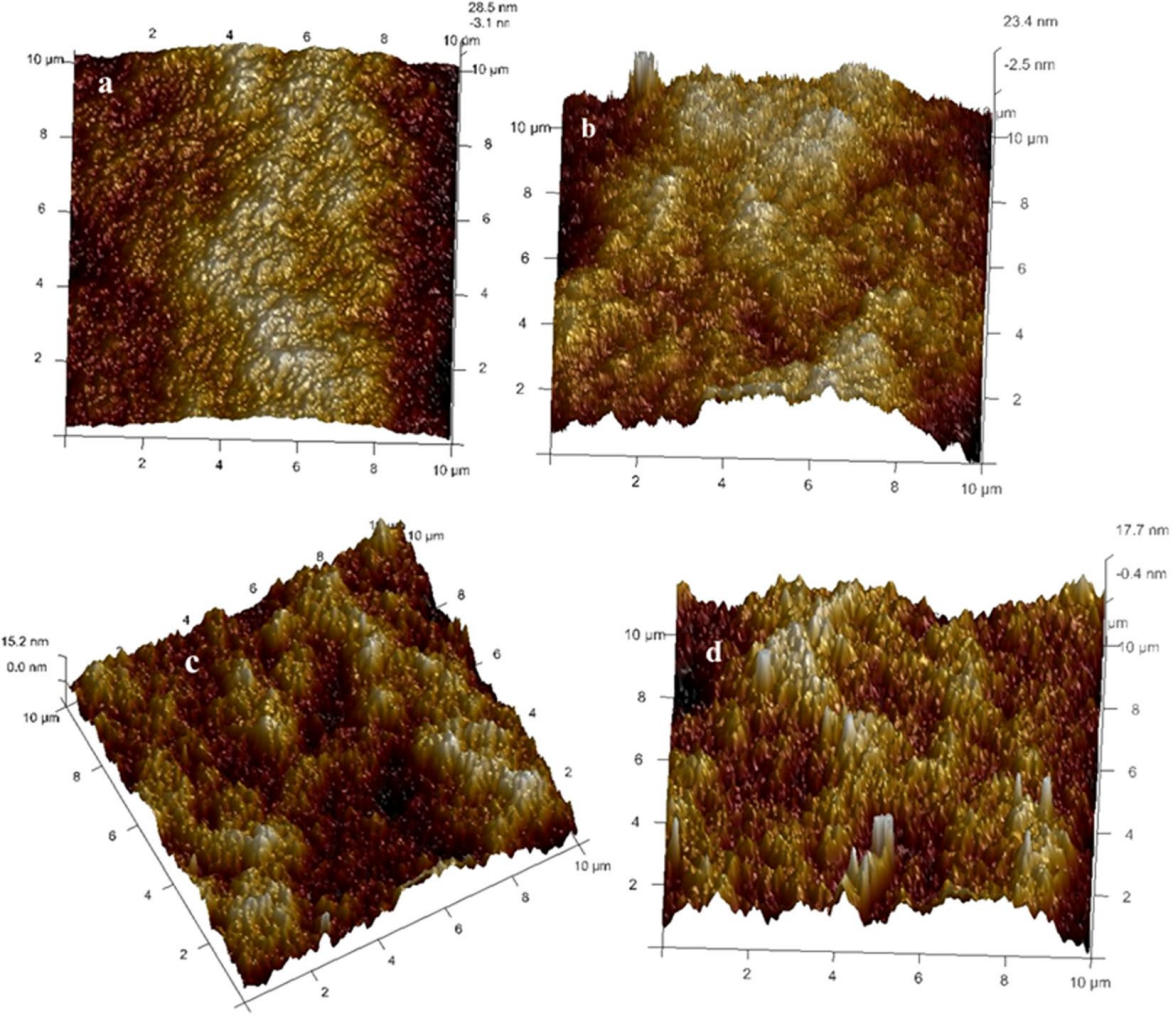
The three dimensional AFM images of (**a**) PEM-0, (**b**) PEM-1, (**c**) PEM-2 and (**d**) PEM-3 membranes.

Figure 9 displays the three dimensional AFM images of the PEI nanocomposite membranes. The roughness parameters of the membrane surfaces were also reported in terms of root mean square of Z data (*Rq*), mean roughness (*Ra*), and mean difference in the height between the highest peaks and the lowest valleys (*Rz*). The results of surface roughness for the prepared membranes were tabulated in the Table 3. From the Table 3 we could observe that roughness parameters of all the modified membranes were higher than that of the PEM-0 membrane. PEM-0 membrane showed root mean square roughness (*Rq*) and mean roughness (Ra) of 7.43 nm and 5.93 nm respectively. Whereas membrane with 3 wt % of modified bentonite exhibited mean roughness (Ra) and root mean square roughness (Rq) of 11.1 nm and 13.9 nm respectively (S-4). According to Zhang *et al*., a membrane with higher surface roughness have higher surface area and causes cavities, thus promoting water permeation rate^28^.

**Table 3:** The roughness parameters of the membranes.

Membrane	Image surface	Surface area	Roughness		
	area (μm^2^)	Difference (%)	*Ra* (nm)	*Rq* (nm)	*Rmax* (nm)
PEM-0	100	0.072	5.93	7.43	67.7
PEM-1	104	1.37	7.13	9.25	99.1
PEM-2	100	0.083	8.71	10.3	108
PEM-3	102	1.62	11.1	13.9	126

### Contact angle measurement

**Figure 10:**
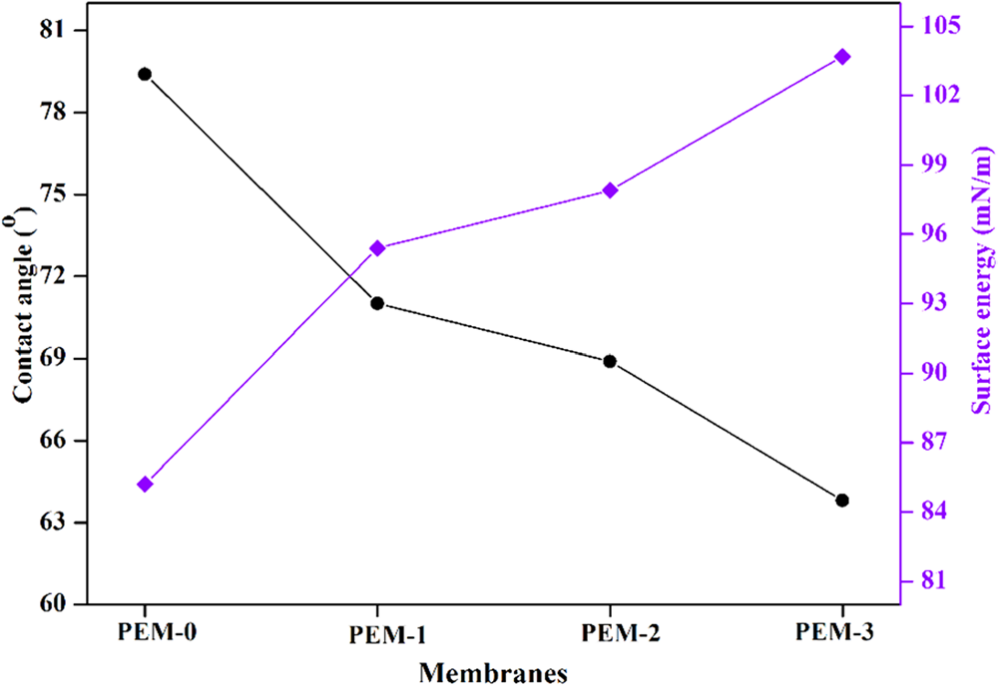
The contact angle and surface energy of the membranes.

**Figure 11:**
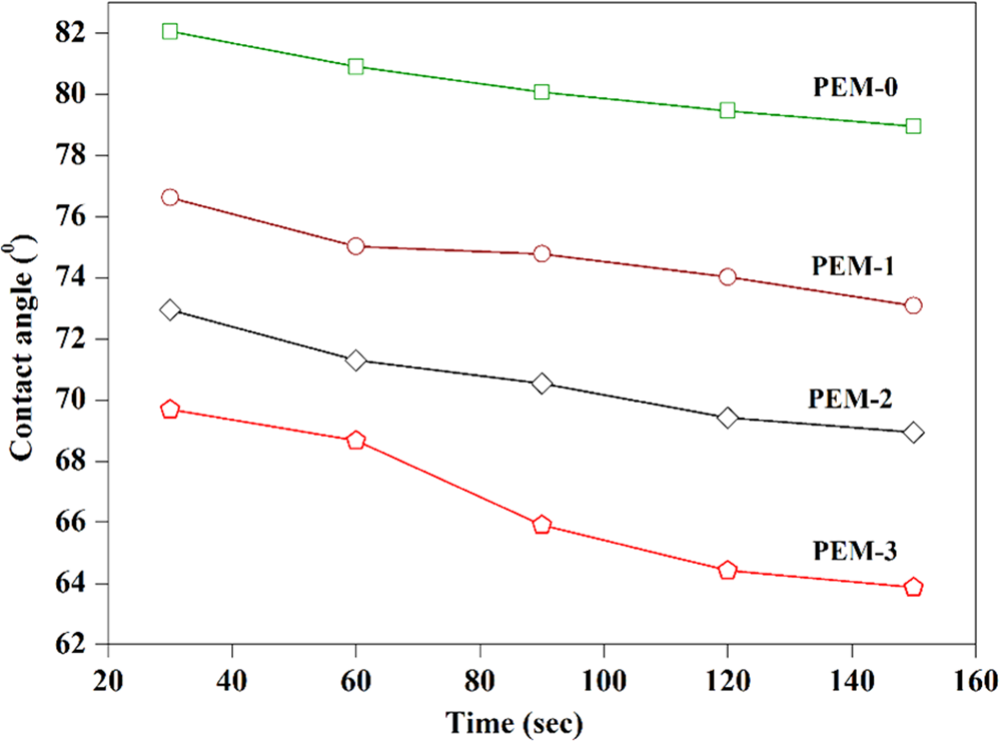
The time dependent contact angle of the membranes.

The surface properties of the membrane have a significant effect on the flux, fouling and other barrier properties. The water contact angle analysis has been generally employed to estimate the surface hydrophilicity of the membrane. The higher contact angle value represents a hydrophobic surface, whereas lower value represents the hydrophilic surface^29^. Figure 10 displays the contact angle and surface energy of the nanocomposite membranes. The decreasing trend of contact angle was observed as increased modified bentonite dosage in the membrane matrix. The membrane with 3 wt. % of additive had shown lowest contact angle of 63.8°, which was nearly 16° less than that of the PEM-0 membrane (S-5). Further, WCA values were used to determine the surface energy (ω_A_) of the resultant membrane. The lowest surface energy of 85.2 mN/m was obtained for the PEM-0 membrane, whereas PEM-3 membrane exhibited highest surface energy value of 103.7 mN/m. These results indicate that, addition of modified bentonite to the membrane matrix could significantly improve the surface hydrophilicity. Furthermore, all the membranes have shown an apparent decline in the contact angle with increasing the drop age. The PEM-0 membrane displayed a slight decrease in the contact angle, whereas modified membranes offered noticeable change with increasing water droplets. At the water dropage of 150 sec, contact angles of the PEM-0, PEM-1, PEM-2 and PEM-3 membranes were 79°, 71°, 68° and 63° respectively (Fig. 11). These results imply that, immobilization of modified bentonite into the membrane matrix not only improve the surface hydrophilicity, but also pore hydrophilicity. The improvement in the membrane hydrophilicity could be substantiated by the fact that during the membrane formation process, hydrophilic modified bentonite spontaneously migrates towards the top surface to reduce the interface energy. Since hydrophilic modified bentonite on the membrane surface could easily interact with water molecules, giving rise to lower contact angle^30^.

### Water uptake capacity and porosity

One more pervasive investigation to understand the bulk hydrophilicity properties of nanocomposite membrane is equilibrium water uptake capacity. This mainly depends on the two factors, firstly on the presence of a number of hydrophilic polar functional groups per unit area of membrane matrix. Secondly, on the membrane morphology i.e, the presence of macrovoids in the membrane sublayers^31^. The water uptake capacities of the prepared membranes were tabulated in the Table 4. The results revealed that, water uptake capacity increases with increased modified bentonite dosage in the membrane matrix. The PEM-0 membrane has shown the lower water uptake value of 34.7%, whereas membrane with 3 wt. % of modified bentonite exhibited up to 71.3%. This confirmed the presence of a hydrophilic additive, which increases the affinity of membrane matrix towards water molecules. Also from the Fig. 7, we could observe that, PEM-3 membrane displayed a more number of macro-voids in the sublayers. This also contributes to the enhanced equilibrium water content of the membranes.

**Table 4:** The properties of the prepared membranes.

Membrane	Thickness (µm)	Porosity (%)	Water uptake (%)	FRR (%)
PEM-0	128	32.4	34.7	23.7
PEM-1	139	42.9	48.2	58.1
PEM-2	143	53.5	65.8	76.5
PEM-3	131	62.7	71.3	84.1

It is well known that, the skin layer of the asymmetric membrane has a major influence on its performance and selectivity. Since the rejection and intrinsic permeation properties are also primarily depend on pore size and average pore size distribution of the membranes^11^. The addition of inorganic additives into the casting solution significantly alters the performance of the resultant membrane. In order to substantiate this, the experimental results showed a noticeable change in the porosity and morphology, this was due to the presence of different amount of modified bentonite dosage. The porosity of all prepared membranes was tabulated in table-3. It can be observed that, the membrane with 3 wt. % of modified bentonite exhibited higher porosity up to 62.7%, while at the same time PEM-0 showed porosity of 32.4%. This change in the porosity could be explained on the basis of influence and migration behaviour of modified bentonite content during the membrane formation process. The addition of modified bentonite to the membrane dope enhances the influx rate of non-solvent (water) and delayed the exchange process between the solvent in polymer dope and non-solvent in the coagulation bath. This improves the ratio of water content in the nascent membrane, increase the porosity. In addition, a portion of PVP was leached out of casting film during the phase inversion and acted as pore former^29^.

### Permeation properties

**Figure 12:**
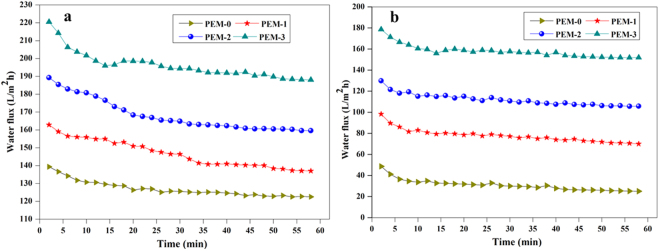
The (**a**) time dependent pure water of the membranes and (**b**) water fluxes of the membranes after BSA protein solution filtration.

The porosity and hydrophilicity are the two important factors, which decides the permeation properties of the membranes. Figure 12a illustrate the time dependent pure water fluxes of the membranes with different amount of modified bentonite content. Initially, a gradual decline in hydraulic permeation rate was observed for all the prepared nanocomposite membranes during compaction. This was probably due to mechanical deformation or compression of membrane structure under the different transmembrane pressure (TMP) during the operation. From the experimental observation, the inclusion of modified bentonite additive in the membrane matrix has a synergistic effect on the physicochemical and hydrodynamic permeation behaviour of nanocomposite membrane. Since membrane with 0 wt. % of modified bentonite showed PWF of 121 L/m^2^h, whereas 3 wt. % of modified bentonite exhibited PWF of 211 L/m^2^h. The water fluxes of the PEM-1, PEM-2 and PEM-3 are 1.33, 1.52 and 1.74 times higher than that of PEM-0 membrane respectively. Here, the pure water fluxes of the nanocomposite membrane showed a similar trend that of porosity and hydrophilicity. This is due to the fact that, the modified bentonite immobilised PEI membrane have a synergistic effect on permeation properties, and the enhanced hydrophilicity can attract the water molecules, facilitating them to pass through the membrane. Furthermore, the structural or morphological changes in the composite membrane compared PEM-0 membrane also influence the PWF. When modified bentonite content increases from 1 wt. % to 3 wt. %, the membrane surface porosity, hydrophilicity, pore interconnection and macrovoid increased. Which significantly reduce the hydraulic resistance and thus improved membrane permeability^28,32^.

### Antifouling properties

The adsorption or deposition of organic foulants particularly protein molecules on membrane surface and pores is the prevalent reason for membrane fouling. It could reduce the membrane flux either temporarily or permanently. The adsorbed protein molecules might cause reversible membrane fouling, which can be easily eliminated by simple water flushing, while flux reduction was caused by irreversible membrane fouling that could not be easily removed^33,34^. BSA protein solution is generally employed as a model protein to examine fouling dynamics of membranes. Figure 12b illustrate the water flux of the membranes after BSA solution filtration. It was observed that, hydraulic permeability rate of the membranes decreased prominently when pure water was substituted by protein solution in the filtration tank, which represents the membranes fouling. After washing the membranes, the pure water flux of the PEM-0 membrane had shown a large decrease. However, the flux reduction for the modified bentonite embedded membranes was lower.

Table 4 represents the % of FRR of all the prepared membranes. The higher FRR indicates better hydraulic cleaning property of the membranes. The flux recovery ratio of the PEM-0 membrane was about 28.7%, indicating a poor self-cleaning property, while it reached up to 58.1%, 76.5% and 84.1% for the PEM-1, PEM-2 and PEM-3 membranes respectively. This change in the flux recovery value of nanocomposite membranes may be attributed to modified membrane comprises of an abundance of negatively charged hydrophilic polar functional groups such as hydroxyl (-OH), carbonyl groups (C=O), and sulfonic acid (-SO_3_H), which impart the hydrophilicity to the membrane surface. Since by diminishing robust hydrophobic-hydrophobic interactions between the membrane surface and foulants. Further, hydrophilic membrane surface is having the capacity to attract water molecules to form the hydrated layer, which acts as a buffer to the adsorption of foulants^35^. Thus reducing the interactions of protein molecules with the membrane surface or pores and decreasing membrane fouling. On the other hand, the electrostatic repulsive interaction between negatively charged membrane surface and BSA molecule could also aid to enhance the fouling resistance behaviour. The experiential outcome is reasonably analogous to the polymer membrane comprising of hydrophilic inorganic additives such as graphene oxide (GO), TiO_2_, SiO_2_ nanoparticles, multiwalled carbon nanotubes (MWCNTs) and other nanoplates^25,32,34^.

**Figure 13:**
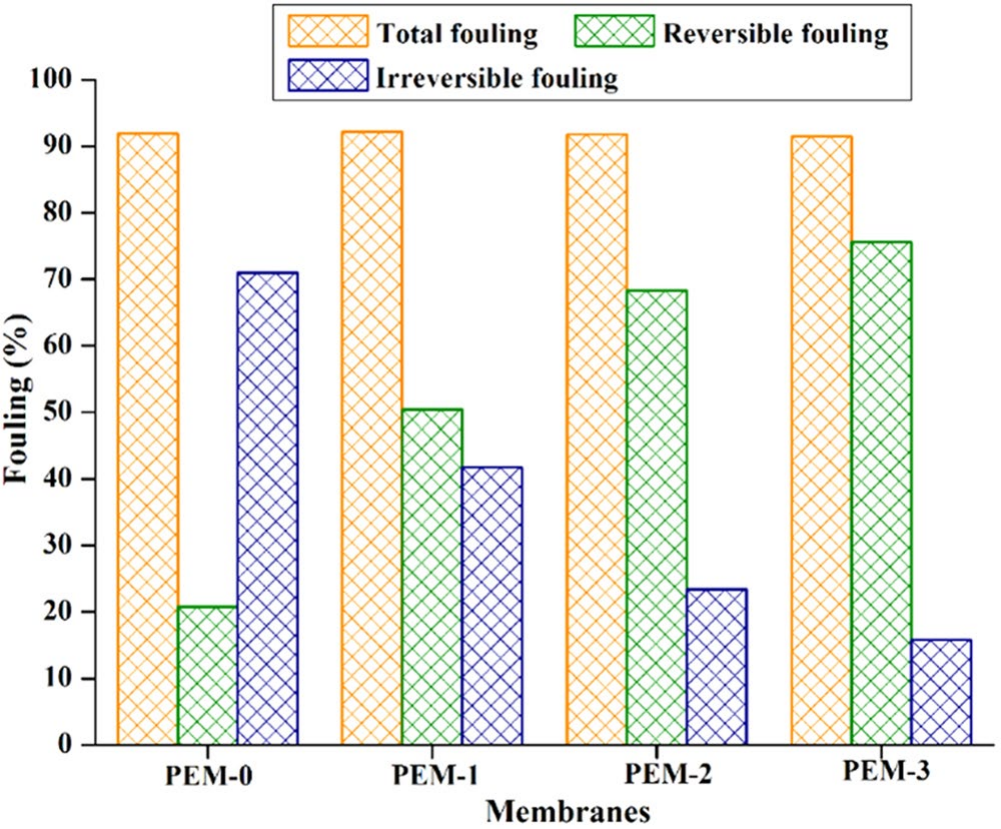
The fouling resistance behaviour of prepared membranes.

Further to examine more about the fouling behaviour, the properties such as total fouling ratio (*R*_*t*_), irreversible (*R*_*ir*_) and reversible (*R*_*r*_) fouling resistance ratios were determined. The fouling nature of the modified membrane after hydraulic cleaning was depicted in Fig. 13. The total fouling ratio (*R*_*t*_) of 92.1% was observed for the PEM-0 membrane. PEM-3 membrane exhibited reversible fouling (*R*_*r*_) up to 75.6% with flux rate of 168.7 L m^−2^h^−1^. After modification, the irreversible fouling percentage in the total fouling drastically dropped to 41.8%, 23.4% and 15.8% for PEM-1, PEM-2 and PEM-3 membranes respectively (S-6). This confirmed that, antifouling nature of hybrid membranes were significantly improved.

### Humic acid rejection properties

**Figure 14:**
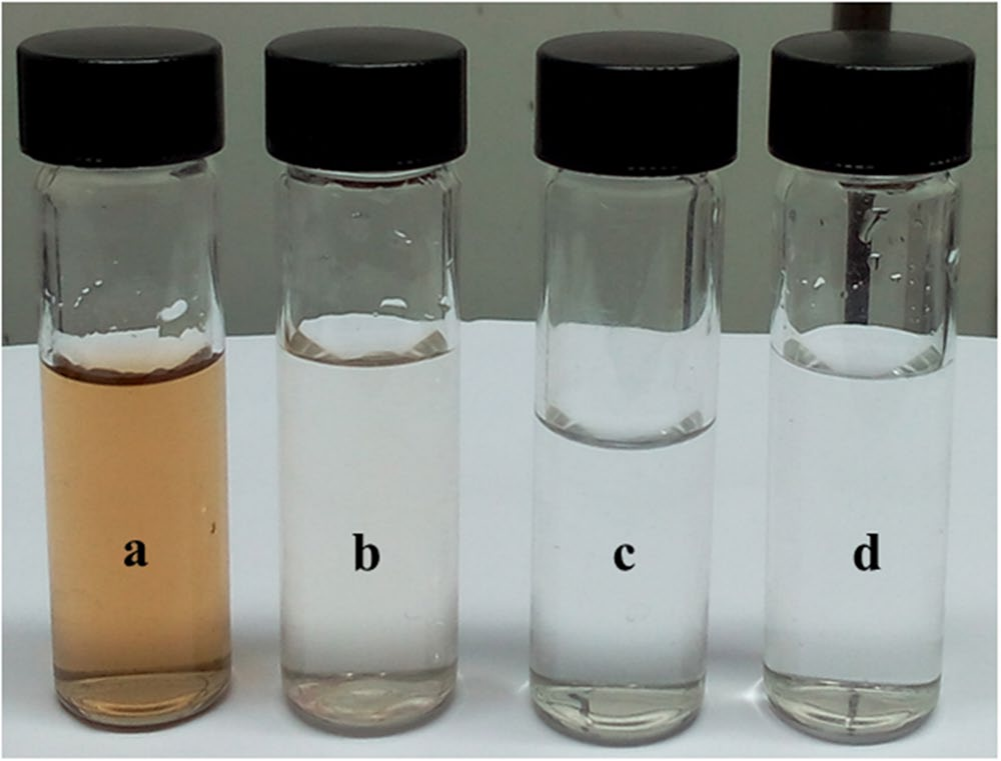
The photographic image of (**a**) HA solution in the feed, (**b**), (**c**) and (**d**) are permeate of PEM-1, PEM-2 and PEM-3 membranes respectively.

**Figure 15:**
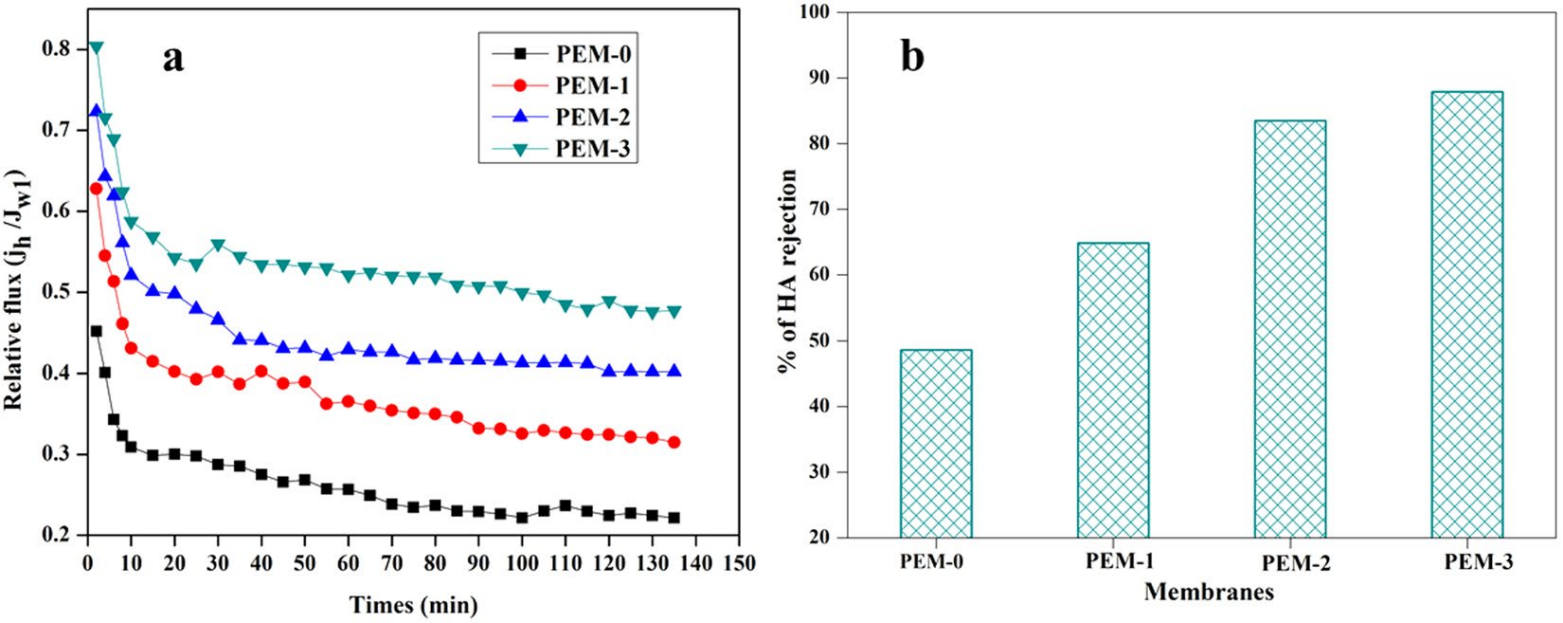
(**a**) The relative flux ratio during HA filtration and (**b**) HA rejected by the membranes at 0.3 MPa TMP.

Since in the current work, to further explore the humic acid rejection and anti-organic fouling property of modified membranes, the filtration experiments were carried out at 0.3 MPa TMP with 200 ppm HA solution (Fig. 14). The flux of all the membranes decreased rapidly at the beginning of each filtration experiment indicates the deposition or adsorption of HA molecules on the membrane surfaces. The permeation behaviour of the membrane during the experiment was measured in terms of flux relative to the initial PWF and results were presented in Fig. 15a. The inclusion of hydrophilic modified bentonite enhanced the relative flux indicating that, higher resistance towards fouling. The membrane without additive showed ~ 24% relative to initial PWF, whereas PEM-3 membrane exhibited highest relative permeate flux of ~60%. The Fig. 15b represents the HA rejection behaviour of the different membranes. From the results, we could observe that, maximum fouling resistance offered by PEM-3 membrane with HA rejection efficacy of 87.6%. It was interesting to note that, foulants on the modified nanocomposite membrane surface can be easily eradicated by simple hydraulic cleaning. Since adsorption or deposition of foulants on membrane surface is considered to be the prominent fouling mechanism and is strongly depending on the physicochemical and structural properties of both membranes and foulants^36,37^. The modified bentonite additives impart the negative charge density and improved hydrophilicity of resultant membrane, thus reduce strong interaction between membranes surface and HA molecules^37,38^.

### Heavy metal ion rejection

**Figure 16:**
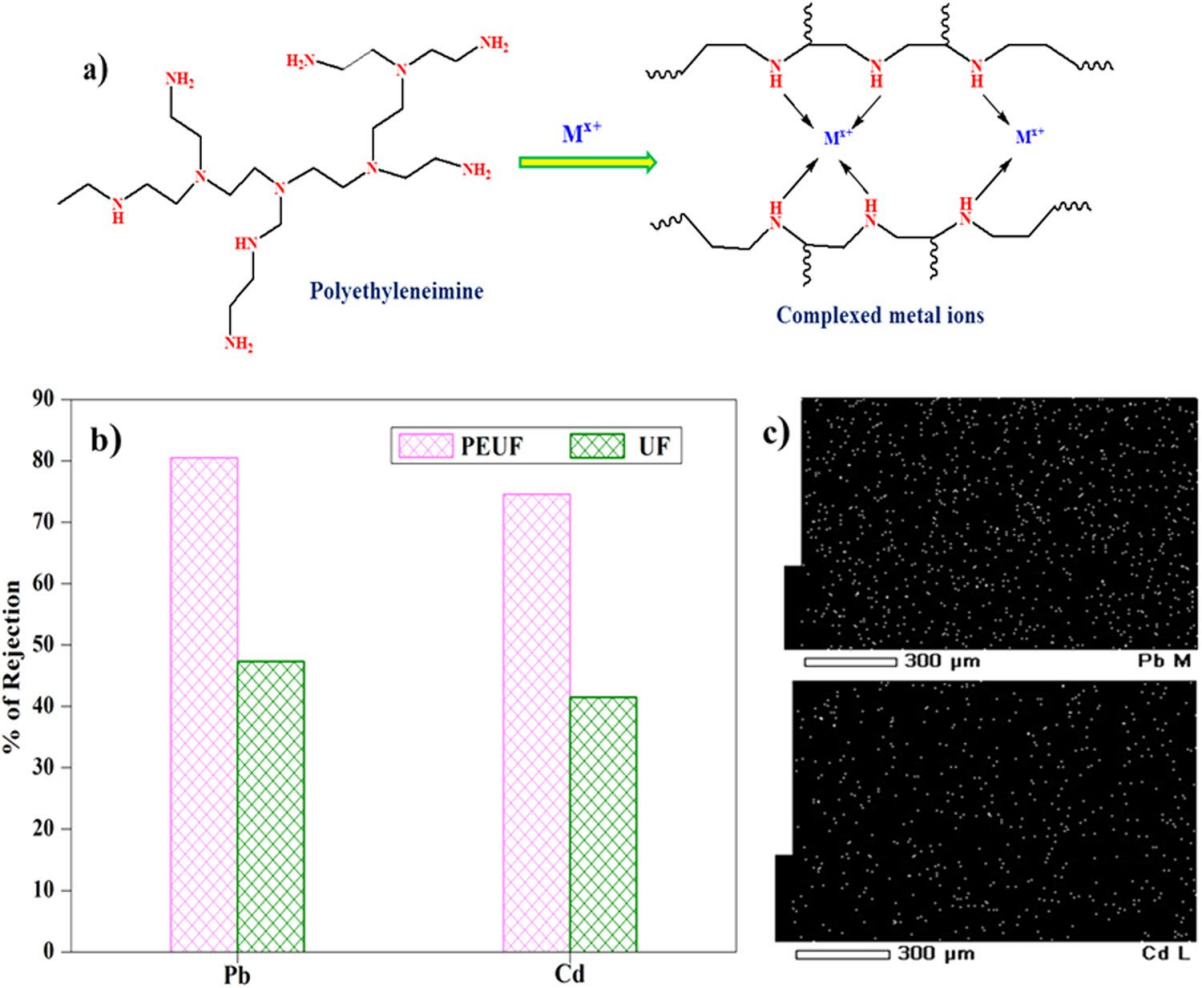
(**a**) The schematic representation of heavy metal ion complex with the complexing agent, (**b**) heavy metal ion rejected by the PEM-3 membrane and (**c**) The elemental mapping of PEM-3 membrane after the heavy metal ion filtration.

UF membranes have also been exploited for removal of toxic and detrimental heavy metal ions from aqueous streams. The prepared nanocomposite membrane was subjected to heavy metal ion rejection experiment under both PEUF and UF filtration process. Among the fabricated membranes, the well performed PEM-3 membrane, which exhibited lower contact angle, highest FRR and good permeability rate was taken for the rejection analyses. The Fig. 16a represents the % of heavy metal ions (Cd^2+^and Pb^2+^) were rejected by the membrane via UF and PEUF method. The mechanism of heavy metal ion compensation Polyethylenimine was presented in Fig. 16b. The membrane showed PEUF rejection efficacy up to 80.5% and 74.6% for Pb^2+^ and Cd^2+^ ions respectively. From the results, we can observe that, % of rejection of the Pb^2+^ was higher than that of Cd^2+^ ions. This might be due to Pb^2+^ forms the larger metal chelate size compared to Cd^2+^ ions. Because Pb^2+^ ion has a smaller size (atomic radius of Pb –180 pm) and having higher electronegativity (2.33) compared to Cd^2+^ ions (atomic radius of Cd–220 pm and electronegativity of 1.69)^39^. The % rejection of Pb^2+^ and Cd^2+^ ions during the UF process was 47.3% and 41.5% respectively. This was due to the heavy metal ion adsorption capacity of the modified bentonite in the mixed matrix membrane. The Fig. 16c represents the elemental mapping analysis of cadmium and lead after filtration experiment. This confirms the interaction of heavy metal ions with the active surface of the membrane. From the experimental analysis, it was observed that PEUF process showed higher % of heavy metal ion rejection compared to normal ultrafiltration process. This was due to, the complexed metal ions have considerably larger size than the membrane pore size. In case of normal UF process, the most of the heavy metal ions are certainly pass through the membrane. Only metal ions adsorbed by the active sites of nanocomposite membranes account for the % of rejection^40^.

### Conclusions

The natural bentonite clay bearing the poly (4-styrenesulfonate) brushes were synthesised by distillation-precipitation polymerisation and employed as performance modifying agent in membrane matrix. The membrane was fabricated by incorporating different amounts of modified bentonite dosage via phase inversion method. The proper immobilisation of additive into the nanocomposite membrane was confirmed by elemental mapping. The modified membrane had shown significant changes in the morphology, porosity, water uptake capacity and hydrophilicity compared to the PEM-0 membrane. The permeation experiment revealed that, pure water flux of the membrane improved to 211 L/m^2^h with 3 wt % of additive dosage. The modified bentonite brushes have a significant influence on anti-organic fouling nature of the membrane. The membrane with 3 wt % of additive dosage has shown FRR value of 84.1% with irreversible fouling ratio of 15.8% and reversible fouling (*R*_*r*_) up to 75.6%. The HA rejection study revealed that PEM-3 membrane having rejection efficiency up to 87.6% and foulants can be easily removed by simple hydraulic cleaning. Moreover, modified membranes were also having the capacity to adsorb heavy metal ion from the aqueous solution. Thus it can be concluded that, the natural bentonite clay bearing poly (4-styrenesulfonate) brushes could be considered as potential candidate to improve the overall membrane performance.

## Electronic supplementary material

Supplementary information
