# Supplementary material for: Removal of metal ions and humic acids through polyetherimide membrane with grafted bentonite clay
Source: Sci Rep. 2018 Mar 16;8:4665. doi: 10.1038/s41598-018-22837-1 (PMC5856751; doi:10.1038/s41598-018-22837-1)
Supplement: Supplementary file 1 — Supplementary information [file 41598_2018_22837_MOESM1_ESM.docx]

**Removal of metal ions and humic acids through polyetherimide membrane with grafted Bentonite clay**

Raghavendra S. Hebbar^1^, Arun M Isloor^1^*, Balakrishna Prabhu^2^, Inamuddin^3,4*^, Abdullah M. Asiri^3,4,*^ and A.F. Ismail^5^

**S-1** The chemical structure of polyetherimide polymer

**
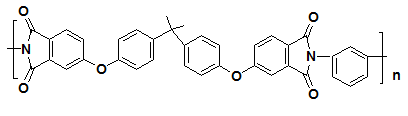
**

**S-2** The representative TEM images of natural bentonite caly


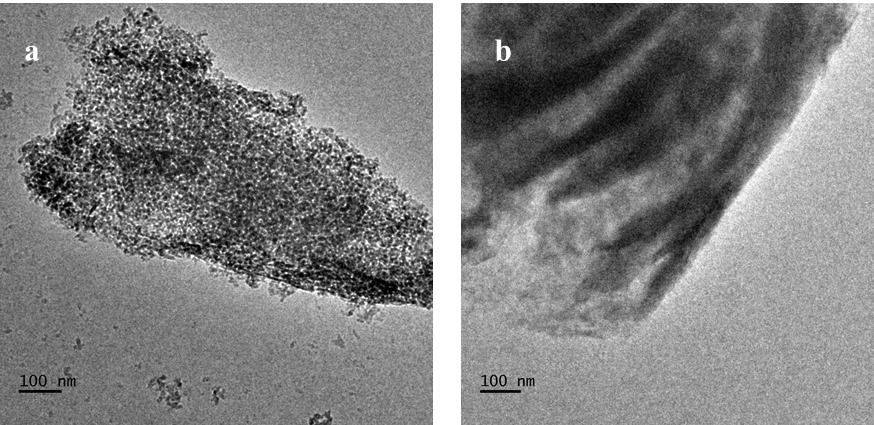


**S-3** Surface SEM images of a) PEM-0 and b)PEM-3 membranes


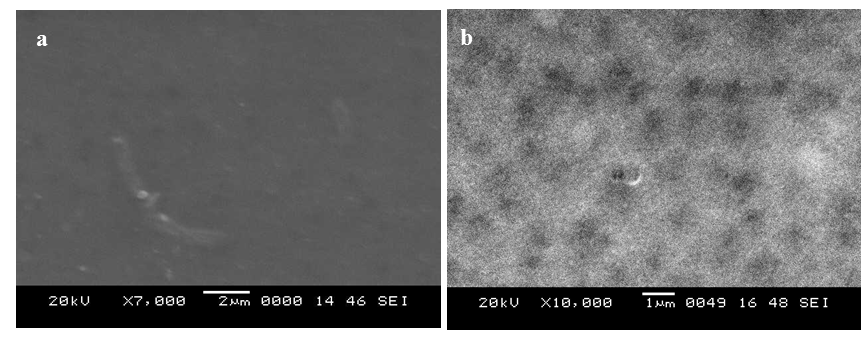


**S-4** The two dimensional AFM images of a) PEM-0, b) PEM-1, c) PEM-2 and d) PEM-3 membranes.


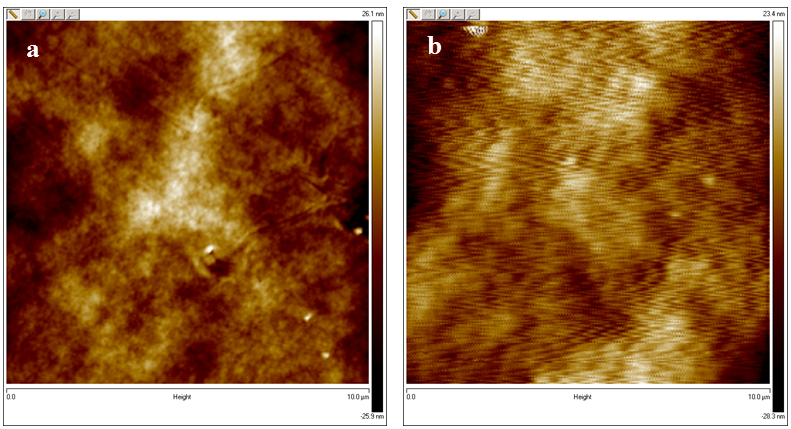


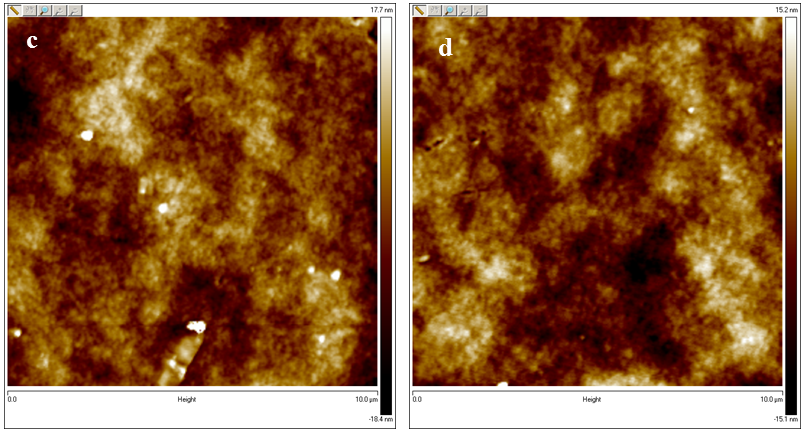


**S-5** The representative contact angle images of a) PEM-0, b) PEM-1, c) PEM-2 and d) PEM-3 membranes.


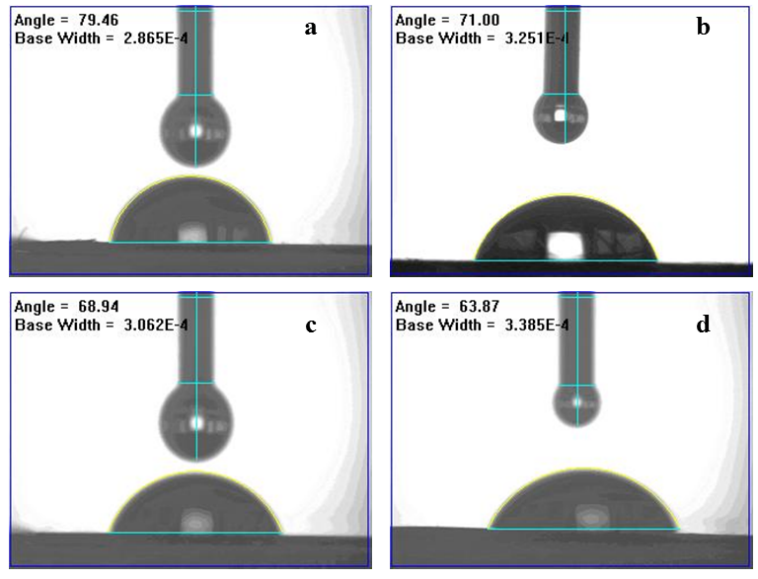


| **Membrane code** | **Permeate Flux (L m^−2^h^−1^)** | | | | **FRR and Fouling recovery (%)** | | | | |
| --- | --- | --- | --- | --- | --- | --- | --- | --- | --- |
|  | $J_{w1}$ | $J_{p}$ | | $J_{w2}$ | $FRR$ | | $R_{t}$ | $R_{r}$ | $R_{ir}$ |
| PEM-0 | 121.6 | | 9.8 | 35.2 | 28.7 | 91.9 | | 20.8 | 71.0 |
| PEM-1 | 162.4 | | 12.6 | 94.5 | 58.1 | 92.2 | | 50.4 | 41.8 |
| PEM-2 | 184.7 | | 15.1 | 141.3 | 76.5 | 91.8 | | 68.3 | 23.4 |
| PEM-3 | 211.6 | | 17.8 | 177.9 | 84.1 | 91.5 | | 75.6 | 15.8 |

**S-6** Filtration and antifouling performance of the membranes

**S-7** The schematic representation of chemical structure of humic acid
